# Treatment effect bias in randomised controlled trials using surrogate outcomes: a preliminary cohort study analysis

**DOI:** 10.1186/1745-6215-12-S1-A73

**Published:** 2011-12-13

**Authors:** Oriana Ciani, Rod S Taylor

**Affiliations:** 1 Peninsula Technology Assessment Group (PenTAG), Institute of Health Services Research, Peninsula College of Medicine & Dentistry, University of Exeter, Exeter, Devon, EX14 3JJ, UK

## Background

Ideally, decisions on the value of health technologies should be based on evidence from well-conducted clinical trials that assess clinically important final patient-relevant outcomes, such as mortality or impaired quality of life. Pressure to reduce the delay in the availability of technologies to patients has led to an increased reliance on the use of surrogate outcomes [1]. A key tenant of surrogate outcomes is unbiased quantification of the predictive treatment effect on the final patient-relevant clinical outcomes. This study compares the treatment outcome of trials that use a surrogate versus a final patient-relevant primary outcome.

## Materials and methods

We searched for all randomized controlled trials (RCTs) published in JAMA, NEJM, Lancet, BMJ, PLoS Medicine and Annals of Internal Medicine in 2005 and 2006 [2]. We distinguished between trials that used a surrogate or a patient-relevant primary outcome. An outcome was defined as a surrogate if it did not directly measure “how a patient feels, functions, or survives” [3] or was judged to be a substitute and predictive of a final outcome [1]. We excluded non-RCTs, composite (of both surrogate and final) outcomes, economic evaluations and non-interventional technologies. Surrogate and final outcome trials were matched on patient population, intervention, journal and year of publication. In this preliminary analysis we compare the two groups of trials based on the statistical significance and direction of their outcome results.

## Results

Of the 639 citations identified by our searches, we included 138 trials that used a primary surrogate outcome (‘surrogate trials’) and 132 trials that used a final patient-relevant outcomes (‘final trials’). Table 1 summarises the trial characteristics used for matching. Other trial characteristics also appeared to be well balanced except for the length of follow-up (i.e. more studies with follow up <30 days and >1 year for final trials).

**Table 1:** Characteristics of included surrogate and final trials.

	Surrogate Trials (N=138) N(%)	Final Trials (N=132) N(%)
*Intervention clinical area*		
Cardiovascular	31(22)	31(23)
Endocrinology	11(8)	4(3)
Gastrology/hepatology	11(8)	11(8)
Infectious disease	28(20)	28(21)
Nephrology/urology	4(3)	4(3)
Neurology	2(1)	3(2)
Obstetrics	5(4)	5(4)
Oncology	4(3)	4(3)
Other	36(26)	36(27)
Pulmunology	6(4)	6(5)

*Population clinical area*		
Cardiovascular	34(25)	31(23)
Endocrinology	14(10)	9(7)
Gastrology/hepatology	9(7)	10(8)
Infectious disease	21(15)	21(16)
Nephrology/urology	2(1)	5(4)
Neurology	2(1)	2(2)
Obstetrics	7(5)	8(6)
Oncology	4(3)	4(3)
Other	36(26)	36(27)
Pulmunology	9(7)	6(5)

*Journal*		
Annals	14(10)	10(8)
BMJ	11(8)	14(11)
JAMA	31(22)	31(23)
Lancet	28(20)	28(21)
NEJM	51(37)	49(37)
PLoS	3(2)	-

*Year*		
2005	64(46)	63(48)
2006	74(54)	69(52)

Surrogate trials were less likely to have adequate evidence of randomisation sequence generation and adopt the ITT principle. We also found clear evidence that final trials were more likely to observe a non-statistically significant (‘neutral’) treatment effect than surrogate trials (49% vs 23%) (Table 2).

**Table 2:** Comparison of outcome results.

	Surrogate Trials (N=138) N (%)	Final Trials (N=132) N (%)	***P-value^a^***
*Study outcome*^b^			*0.006*^c^
Positive	49 (36)	41 (31)	
Negative	5 (4)	4 (3)	
Neutral	32 (23)	65 (49)	

*Risk of bias*			
Statement of ITT	95 (69)	106 (80)	*0.031*
Automated sequence generation	82 (59)	93 (70)	*0.058*
Allocation concealment	96 (70)	97 (63)	*0.476*
Blinding/Placebo	72 (52)	60 (45)	*0.27*

^a^Chi-square test, unless otherwise specified.

^b^‘positive’: treatment group superior to control (P≤0.05); ‘negative’, control group superior to treatment (P≤0.05); ‘neutral’, treatment and control indifferent, P>0.05).

^c^Fisher’s exact test. Multiple-interventions trials are excluded from this comparison.

## Conclusions

This preliminary analysis supports our initial hypothesis that the use of surrogate outcomes is more likely to lead statistically significant treatment effects than patient-relevant primary outcomes. We are currently undertaking additional analysis using actual effect sizes in meta-analytic/meta-regression framework. These results have important implications for payers faced with making coverage decisions on the effectiveness and cost-effectiveness of new treatments based on surrogate rather than final clinical trials data.
